# Mechanical Properties of Large Slump Concrete Made by Post-Filling Coarse Aggregate Mixing Procedure

**DOI:** 10.3390/ma13122761

**Published:** 2020-06-18

**Authors:** Jinqing Jia, Qi Cao, Lan Luan, Ziyi Wang, Lihua Zhang

**Affiliations:** State Key Laboratory of Coastal and Offshore Engineering, School of Civil Engineering, Dalian University of Technology, Dalian 116024, China; jiajq@dlut.edu.cn (J.J.); 18841109263@139.com (L.L.); ziyiwang@mail.dlut.edu.cn (Z.W.); lihua2018@dlut.edu.cn (L.Z.)

**Keywords:** coarse aggregate interlocking, post-filling coarse aggregate, mechanical properties, post-filling coarse aggregate ratio

## Abstract

High-performance pumping concrete has been widely used in high-rise building construction because of its superior qualities. However, early cracking problems can occur in cast-in-place pumping concrete, which is due to the excessive use of cement. In this paper, an innovative concrete mixing procedure called “post-filling coarse aggregate concrete” (PFCC) was adopted and applied to large slump concrete. The influence of the post-filling coarse aggregate (PFA) ratio on the mechanical properties of large slump concrete were investigated. Three different concrete strength grades (C30, C40 and C50) and five different PFA ratios (0%, 10%, 15%, 20%, 25%) were considered. The designed slump for the reference concrete was 180–200 mm. Experimental tests on the compressive strength of cubic specimens, axial compressive strength of prism specimens, splitting tensile strength, modulus of rupture and modulus of elasticity were performed. All tests were conducted in room conditions. Results showed that the slump of the PFCC decreases as the PFA ratio increases. It also indicated that, in general, the mechanical properties of PFCC are not linearly increasing as the PFA ratio rises, and there exists a turning point. Based on the experimental investigation and analysis in this study, the optimum post-filling coarse aggregate ratio is recommended to be 20%.

## 1. Introduction

Coarse aggregate interlocking concrete is a new type of concrete mixing process, designed to replace a portion of cementitious materials with low cost coarse aggregate to maximize the ratio of aggregate to binder (coarse aggregate to cement binder ratio). The conventional concrete with the same water-cement ratio as coarse aggregate interlocking concrete is made usually as reference concrete (RC). Compared with reference concrete, the coarse aggregate interlocking concrete increases the amount of coarse aggregate and reduces the amount of cementitious materials. It not only improves the concrete properties such as strength, modulus of elasticity, shrinkage reduction and durability, but also exerts a positive impact on energy conservation and carbon dioxide emission reduction. Recycled coarse aggregate concrete also presents its potential for economic and environmental sustainability and has been studied by researchers extensively [1,2,3,4,5]. Researchers have studied the coarse aggregate interlocking concrete [6,7,8], and it has been successfully applied to hydraulic dam engineering [6] and pavement engineering [9] as well as ultra-high-performance concrete (UHPC) [10,11]. In recent years, researchers have started to investigate the basic properties performance of coarse aggregate interlocking concrete [12,13] including scattering-filling coarse aggregate concrete [14,15].

Concrete is a multi-phase material composed of coarse aggregate, mortar and an interfacial transition zone between the two. As the skeleton of concrete strength, coarse aggregate is usually the strongest element in concrete with the best volume stability and durability [16,17]. Meddah et al. [18] found that the coarse aggregate content has a great influence on the compressive strength of ordinary concrete and high-strength concrete. It indicated that the compressive strength of ordinary concrete increased with the increase of coarse aggregate content at certain range. However, it decreased after the coarse aggregate reached a certain value. Meanwhile, Stock et al. [19] reported that when the volume content of coarse aggregate is 20%, the compressive strength and tensile strength of concrete were both lower than that of cement mortar. When the content reached 40%, the compressive strength and tensile strength reached their lowest. It exhibited that the compressive strength and tensile strength began to increase significantly until the value reaches 60%. The increase of coarse aggregate content not only improved the concrete strength, but also improved the other properties of the concrete. Counto [20], Hirsch [21], Tasdemir and Karihaloo [22] found that the modulus of elasticity, effective stress intensity factor, fracture energy and splitting tensile strength of concrete increased with the increase of coarse aggregate content. At the same time, it showed that the improved dosage of the coarse aggregate and the lower content of the cementitious material reduced the shrinkage deformation of the concrete, effectively solving the early cracking problem and improving the durability of the concrete. In addition, coarse aggregate is the cheapest component in concrete other than water. Increasing the amount of coarse aggregate could reduce the cost of concrete material.

Post-filling coarse aggregate concrete is one kind of coarse aggregate interlocking concrete. The coarse aggregate at different gradations is evenly added to premade concrete before the pumping and casting. The strength of the coarse aggregate itself is much higher than that of the mortar, and the strength of the concrete can be enhanced within a certain volume content range of coarse aggregate. Shen et al. [23,24] studied the so called “scatter-filling coarse aggregate concrete” experimentally and found that the coarse aggregate interlocking can be achieved by scatter filling coarse aggregate technology for either high strength concrete or self-consolidating concrete. It indicated that as the volume replacement ratio of coarse aggregate increased from 0% to 30%, the strength and modulus of elasticity of the concrete reached the maximum as the volume ratio of coarse aggregate reached 20%. Meanwhile, the chloride permeability coefficient and the dry shrinkage coefficient of concrete decreased with the increase of the volume ratio of coarse aggregate. The study by Shen et al. [25] also demonstrated that the coarse aggregate in the scatter filling coarse aggregate concrete can be effectively interlocked, so that the aggregate can fully exert the strength skeleton function in the concrete. In addition, SEM results [25] showed that the interfacial bonding between the scatter filling coarse aggregate and the cement paste was tighter than bonding between the aggregate and cement paste in ordinary concrete. It stated that due to the dry surface of coarse aggregate, the water can be adsorbed into the aggregate pores and the water-cement ratio of the interface transition zone was reduced, which caused the increase of density of the interface transition zone and enhancement of concrete strength.

Shen et al. [26] investigated the concrete composition and economic indicators by comparing different construction technology. It concluded that the concrete prepared by the scatter filling coarse aggregate process presented the highest strength and it was significantly improved compared with the concrete made by conventional process. It found that the water cement ratio and shrinkage of scatter filling coarse aggregate were much lower than that of conventional concrete. It also concluded that, by economic and environmental analysis, the scattering filling coarse aggregate concrete reduced the production cost as well as the environment impact of producing concrete.

It is well known that the interfacial transition zone between the coarse aggregate and cement paste is very critical and could determine the failure of concrete. Normally, the following measures were adopted to improve the strength and mechanical performance in the interfacial transition zone (ITZ): (1) decrease water/cement ratio; (2) use supplementary cementitious materials or high performance admixture; (3) select the high quality aggregate to improve the ITZ performance; (4) improve the concrete fabrication process technology, i.e., mixing, placement and curing technology. Among all the measures mentioned above, the investigation of the effect of the mixing procedures on the mechanical performance of concrete is the objective in this study, by using post-filling coarse aggregate concrete. The post-filling coarse aggregate concrete technology not only realizes the green high-performance of concrete, but also significantly reduces the cost of concrete and reduces carbon-dioxide emissions, which is one of the effective ways to achieve low-carbon emissions in the cement concrete industry. Although Shen et al. [14,15,23,24,25] has studied the concrete properties obtained by the scattering-filling coarse aggregate process, the high slump concrete made by post-filling coarse aggregate technology has not been reported on.

The study of the basic mechanical properties of post-filling coarse aggregate is crucial to understanding its structural behavior in order to facilitate its application in engineering. Hence, the post-filling coarse aggregate concrete (PFCC) with different post-filling ratios and different concrete strengths are studied in this paper. In this study, the mixture design and optimization for post-filling coarse aggregate large slump concrete will be performed for different concrete strength grades. The slump loss, compressive strength of cubic specimen and prism specimen, splitting tensile strength, modulus of elasticity as well as modulus of rupture are studied. Through the experimental study, the relationship between the post-filling coarse aggregate (PFA) ratio and the above properties of large slump concrete are obtained, and the optimized PFA ratio is recommended. The percentage of the volume of the coarse aggregate to the total volume of the concrete is defined as post-filling coarse aggregate ratio. Five PFA ratios (0%, 10%, 15%, 20%, 25%) and three different concrete grades (C30, C40 and C50) are studied as parameters in this study. The PFA ratio of 0% indicates the reference concrete without post filled coarse aggregate. It aims to provide research data and design guidelines for the application of post-filling coarse aggregate concrete in the building engineering as well as civil infrastructure.

## 2. Experimental Program

### 2.1. Materials

**Figure 1:**
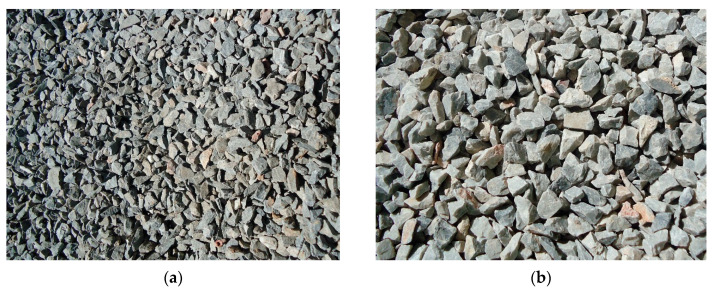
Coarse aggregates: (**a**) for reference concrete; (**b**) for post-filling coarse aggregate concrete.

P·II32.5R cement and P·II42.5R ordinary Portland cement were used. Fly ash was used to replace part of the cement. The coarse aggregate for reference concrete was continuous graded gravel with a particle size of 5–10 mm, as shown in Figure 1a. The coarse aggregate for post-filling was made of 5–16 mm continuous grade limestone gravel with the apparent density of 2500 kg/m^3^, as shown in Figure 1b. The sand was made of ordinary natural river medium sand. Polycarboxylate acid superplasticizer ViscoCrete3301 was used with molecular weight of 5000–50000 provided by local dealer of Sika Company (Dalian, China). The technical properties of superplasticizer are shown in Table 1.

### 2.2. Mixture Proportions

The design of the mixture proportions of concrete is to meet the workability and strength requirements. Three different compressive strength (30 MPa, 40 MPa and 50 MPa for cubic specimens) of concrete were designed in this study, which is denoted as C30, C40, and C50. The required slump of concrete in this study is 180–200mm. The mixture proportions are shown in Table 2.

### 2.3. Mixing Procedures for Post-Filling Coarse Aggregate Concrete

**Figure 2:**
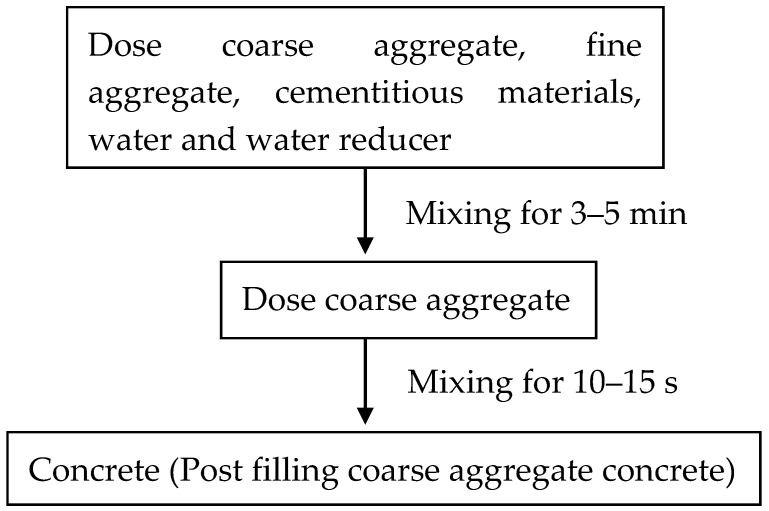
Flow diagram for mixing procedure of post-filling coarse aggregate concrete.

Before mixing the concrete, the inside of the mixer was prewetted. Then the pre-weighed materials including coarse aggregate, fine aggregate, cementitious materials and water were poured into the mixer in turn. The polycarboxylic acid superplasticizer was dissolved into the water and added into the mixture. All mixture components were mixed in a self-falling mixer for 3 to 5 min. After that, the coarse aggregate was filled into the concrete mixture and then was stirred for 10 to 15 s. Finally, a slump test was performed for the fresh concrete and specimen’s mechanical properties. After the specimens were cured for 48 h in the room environment (20 ± 2 °C temperature, 60 ± 5% relative humidity), specimens were demolded and immediately moved to the standard curing room for 28 days before testing. The steps of post-filling coarse aggregate are shown in Figure 2.

### 2.4. Specimen Preparations

Specimens for the compressive strength of cubic specimen (*f_cu_*), splitting tensile strength (*f_ts_*), modulus of rupture (*f_r_*), modulus of elasticity (*E_c_*), compressive strength of prism specimen (*f_c_*) were prepared according to the Chinese standard GB/T50081-2002 [27]. Three concrete strength grades (C30, C40 and C50) and five coarse aggregate post-filling ratios (0%, 10%, 15%, 20%, and 25%) were considered in this study. The selection of post-filling ratios was based on the preliminary test. Three specimens were prepared for each strength property. Table 3 shows the specimen sizes.

## 3. Results and Discussion

### 3.1. Slump

**Figure 3:**
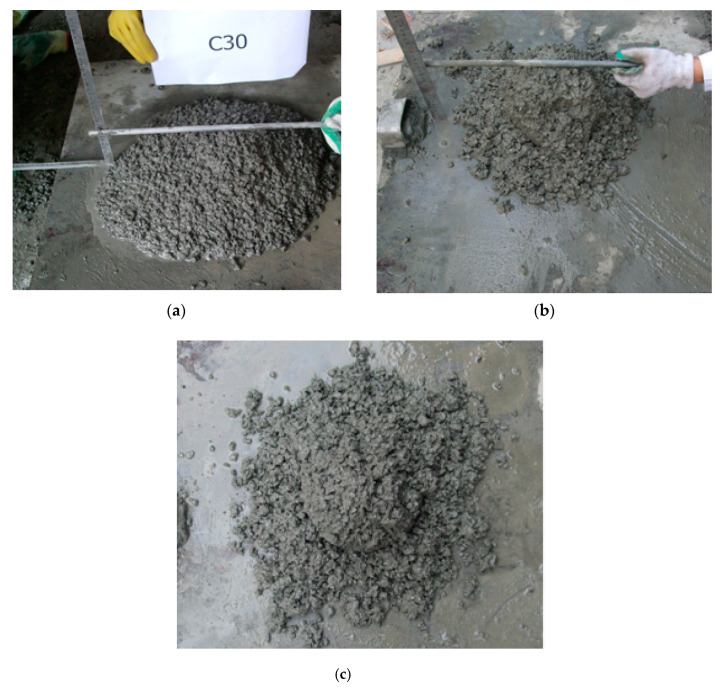
Typical results of slump test: (**a**) reference concrete; (**b**) post-filling coarse aggregate concrete (10% post-filling ratio); (**c**) reduced workability for post-filling coarse aggregate concrete (25% post-filling ratio).

It was observed that, during the slump test, when the post-filling ratio of coarse aggregate is equal to less than 15%, the concrete mixture fell slowly and evenly under the action of gravity. However, when the post-filling ratio is equal or higher than 20%, the workability of the post-filling coarse aggregate concrete was reduced, and the fresh concrete showed a tendency to segregate, which eventually led to collapse on one side of the concrete mixture. Figure 3 shows the typical results of the slump test.

The slump test results are shown in Table 4. As shown, the slump of the post-filling coarse aggregate concrete (PFCC) decreases with the increase of the post-filling coarse aggregate ratio. It also exhibits that when post-filling coarse aggregate (PFA) ratio is not more than 15%, the slump is larger than 140 mm which meets the construction requirements (140 mm is set as target slump in this study). It is believed that the pores and dry surfaces on the coarse aggregate will absorb the water in the concrete and it causes the low slump of high post-filling coarse aggregate ratio.

### 3.2. Compressive Strength of Cubic Specimens

#### 3.2.1. Distribution of Coarse Aggregate

**Figure 4:**
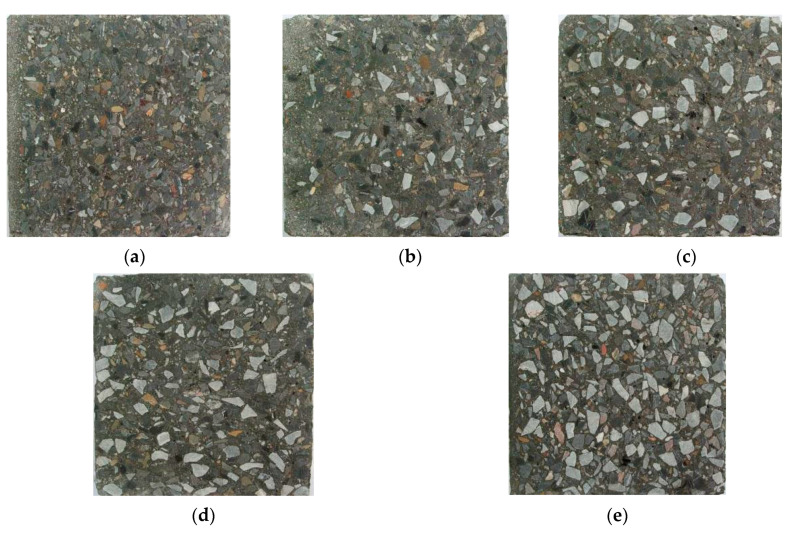
Sections of concrete: (**a**) reference concrete; (**b**) 10% PFA ratio; (**c**) 15% PFA ratio; (**d**) 20% PFA ratio; (**e**) 25% PFA ratio.

Figure 4 shows the distribution of aggregates in the vertical cross section of the concrete specimens with post-filling aggregate ratios of 0%, 10%, 15%, 20% and 25%, respectively.

It can be seen from the Figure 4a that most of the coarse aggregate in the reference concrete is suspended in the mortar, and the strength of the coarse aggregate cannot be fully exerted. From Figure 4b–e, it shows that, simply based on the photo observations, the coarse aggregate content increases and the cement paste content decreases in the cross section with the increase of the post-filling aggregate ratio. It is speculated that, with the increase of the post-filling aggregate ratio, an increase of the connections and interlocking between the coarse aggregate was formed in the cross section.

#### 3.2.2. Failure Mode

**Figure 5:**
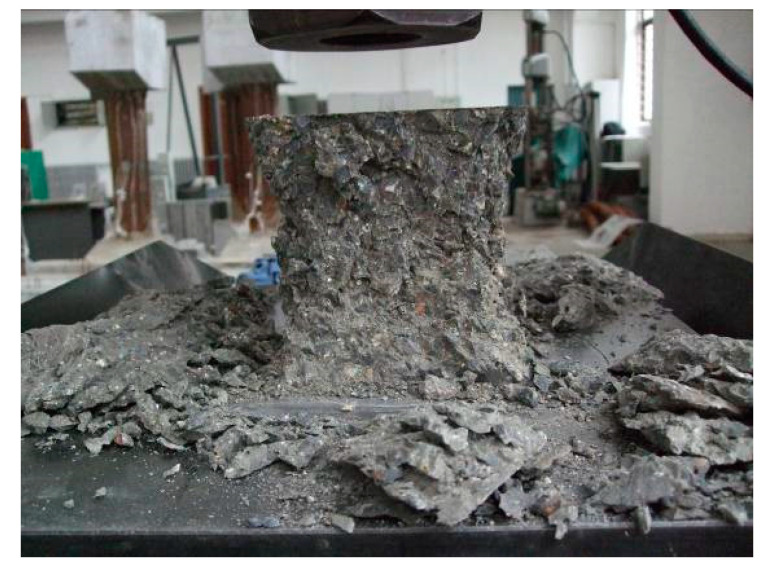
The typical failure mode of cube compressive test for post-filling coarse aggregate concrete.

During the compression test of the cubic specimens, there was no significant difference shown between the failure modes of the reference concrete and the post-filling aggregate concrete. In the initial stage of loading, there was no crack on the surface of the test specimen. As the load was gradually increased, the stress was continuously increased, and cracks in the parallel direction to the load started to appear in the middle of the specimen on the side surface. As the load continued to increase, the cracks gradually developed to the top and bottom of the specimen and new cracks were also generated. In the end, the surface of concrete began to peel off and two cone-shaped failure modes were formed. The failure mode of the cubic specimen is shown in Figure 5.

It was observed that the failure of the low-strength grade PFCC (C30 and C40) mainly occurred at the interface between the coarse aggregate and the cement paste. It is primarily the bond failure between the coarse aggregate and cement paste. While for the C50 grade PFCC, in contrast to the C30 and C40 grade PFCC, the brittle splitting failure of the coarse aggregate was observed.

#### 3.2.3. Compressive Strength Results

**Figure 6:**
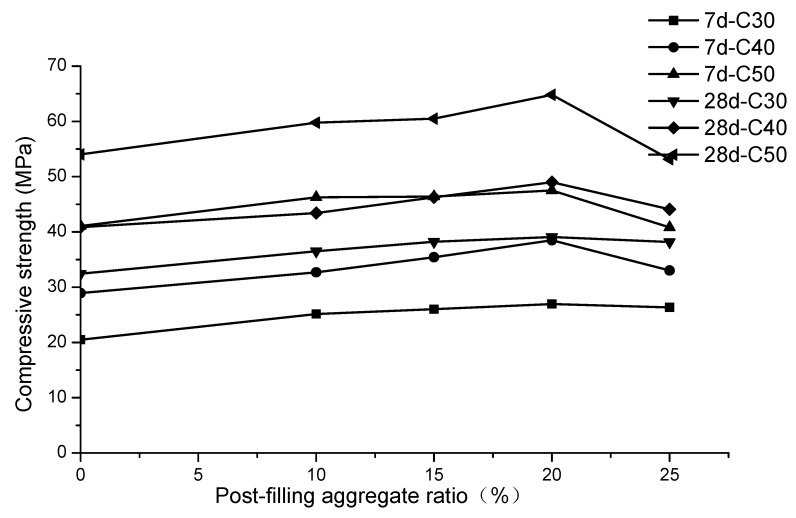
Relationship between post-filling aggregate ratio and cube compressive strength.

The test results of the compressive strength of the cubic specimens are shown in Table 5. The relationship between the compressive strength and post-filling aggregate ratio is shown in Figure 6.

It can be seen from the Figure 6 that the same trends are observed for all three strength grades. The cubic compressive strength first increased and then decreased as the PFA ratio increased for both the 7-day and 28-day strengths. The cubic compressive strength *f_cu_* reached the maximum when the PFA ratio was 20% for the C30, C40 and C50 concrete. The maximum 7-day compressive strength for the C30, C40 and C50 was 26.9, 38.5 and 47.5 MPa, respectively, which increased by 6.4, 9.6 and 6.4 MPa, respectively, compared with the reference concrete. The percentage of compressive strength increase was 31.2%, 33.2% and 15.6%, respectively. Meanwhile, the 28-day maximum compressive strength of C30, C40 and C50 reaches 39.1, 49.0 and 64.8 MPa respectively, increased by 6.7 (20.7%), 8.1 (19.8%) and 10.8 MPa (20.0%), respectively. It is therefore recommended that the optimum post-filling aggregate ratio is 20%. Similarly, in Shen’s study [15], it was reported that the compressive strength of concrete at 28-day reached the maximum at 10% or 15% of scattering filling ratios for either scattering-filling natural or recycled aggregate. It was also shown that the 7-day compressive strength reached the peak at 10% or 15% for scattering filling recycled aggregate [15].

The dry surface of the post-filling coarse aggregate absorbs the water in the reference concrete, reduces the water cement ratio of the newly formed interfacial transition zone (ITZ) in post-filling coarse aggregate concrete and increases the compactness of the new ITZ. Therefore, the strength of the ITZ is enhanced, and at the macro level, the compressive strength concrete is improved compared with the reference concrete. These are the speculated reasons that the compressive strength of the concrete is increased after post-filling aggregate is added.

However, Figure 6 indicates that the 20% PFA ratio is generally the turning point for compressive strength enhancement. This can be explained by the fact that, when the PFA ratio reaches 25%, as mentioned above, the large amount of dry coarse aggregate reduces the workability of the PFCC. It was also observed that a large amount of air bubbles existed in the 25% PFCC. On the other hand, when the PFA ratio is less or equal than 20%, the cement mortar is enough to bond all the aggregate so that the aggregate in the concrete could improve the concrete compressive strength. However, when the PFA ratio is higher than 20%, the extra cement mortar is not enough to cover the post-filling coarse aggregate, which leads to the appearance of internal pores. It was also reported in Shen et al.’s study [14] that it was difficult and took extra time to consolidate the concrete with the 30% scattering-filling coarse aggregate content. In all, the unsatisfactory workability at the large post-filling ratio (20% for cubic compressive strength), which leads to the incompact internal structure of the PFCC, is speculated to cause the turning point of the concrete’s compressive strength.

**Figure 7:**
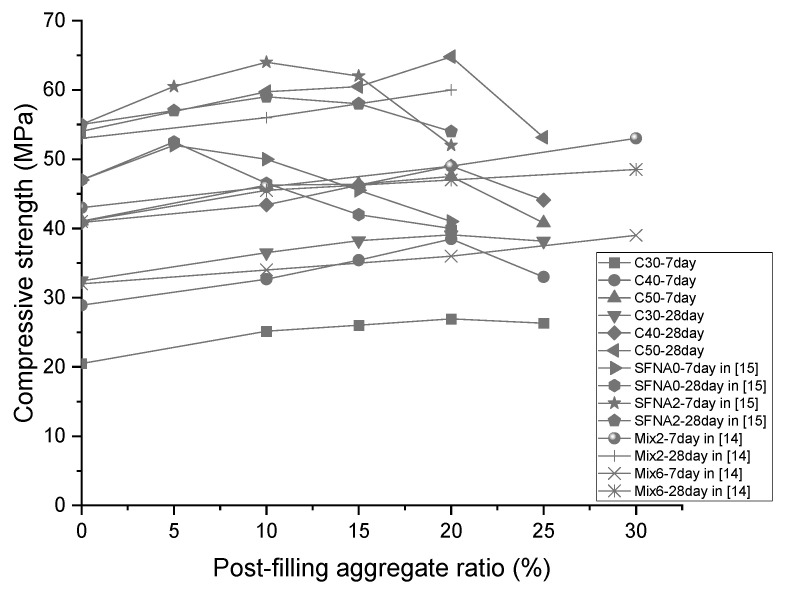
Comparison of compressive strength in this study with literature results.

Meanwhile, the compressive strength of the PFCC in this study was compared with that of scattering filling coarse aggregate concrete in the literature ([14,15]), as exhibited in Figure 7. SFNA0 and SFNA2 indicate the scattering filling coarse aggregate with different natural coarse aggregate gradations, showing that the compressive strength in [15] also increased first and then decreased with the peak value being at 5% and 10% PFA ratio for 7-day and 28-day compressive strength, respectively, which is similar to the results in this study.

### 3.3. Splitting Tensile Strength

**Figure 8:**
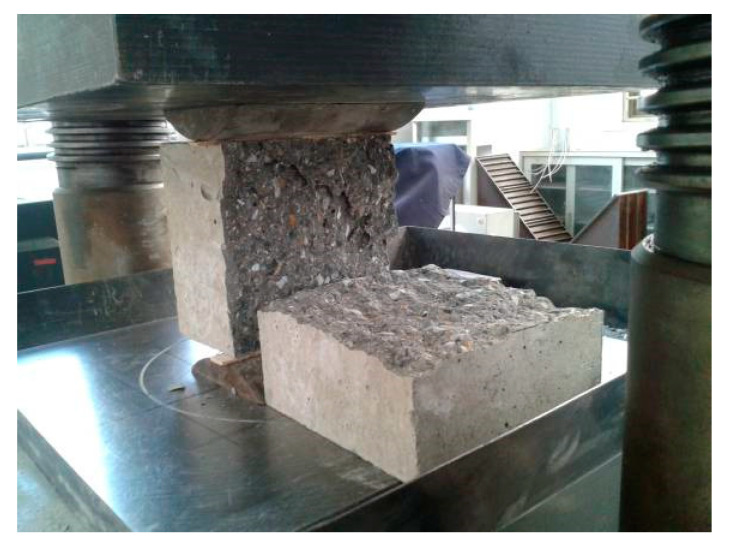
The failure mode of specimen for splitting tensile strength test.

In general, the brittle failure at the splitting tensile strength was observed for all concrete specimens, as shown in Figure 8. Similar to the compressive strength, for the low-strength (C30 and C40) concrete specimens, the cracking failure along the interface between the coarse aggregate and the cement paste was observed. For C50 specimens, the failure was due to the splitting cracking of the coarse aggregate itself. In all, the post-filling aggregate process does not show significant effect on the splitting tensile strength *f_ts_* of concrete.

**Figure 9:**
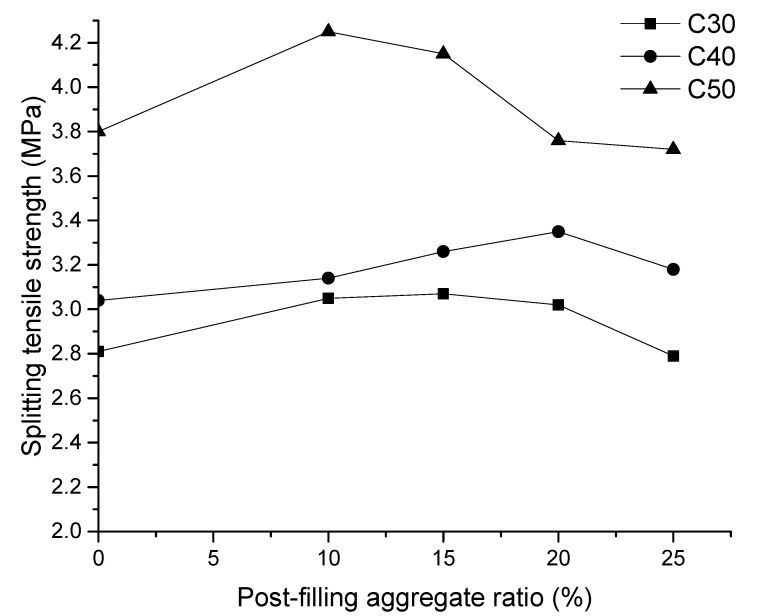
Relationship of post-filling aggregate ratio and splitting tensile strength.

The splitting tensile strength test for the cubic specimen was conducted to obtain the tensile strength of the concrete. The experimental results are shown in Figure 9. As shown, the splitting tensile strength of the PFCC increases and then decreases with the increase of the PFA ratio. There exists a turning point in tensile strength-PFA ratio for all three concrete strength grades. For the C30 grade concrete, the highest tensile strength is 3.07 MPa, which is increased by 0.26 MPa (9.3%) compared with the reference concrete. For C40 concrete, the highest tensile strength is 3.35 MPa, which shows an increase of 0.31 MPa (10.2%) compared with the reference concrete. For C50 concrete, the highest tensile strength reaches 4.25 MPa, which is enhanced by 0.45 MPa (11.8%) compared with the reference concrete. The highest strengths occurred at the PFA ratios of 15%, 20%, and 10% respectively for C30, C40 and C50 concrete. It is suggested that the optimized PFA ratio for PFCC is between 10% and 15% for the best of splitting tensile strength.

### 3.4. Modulus of Rupture

**Figure 10:**
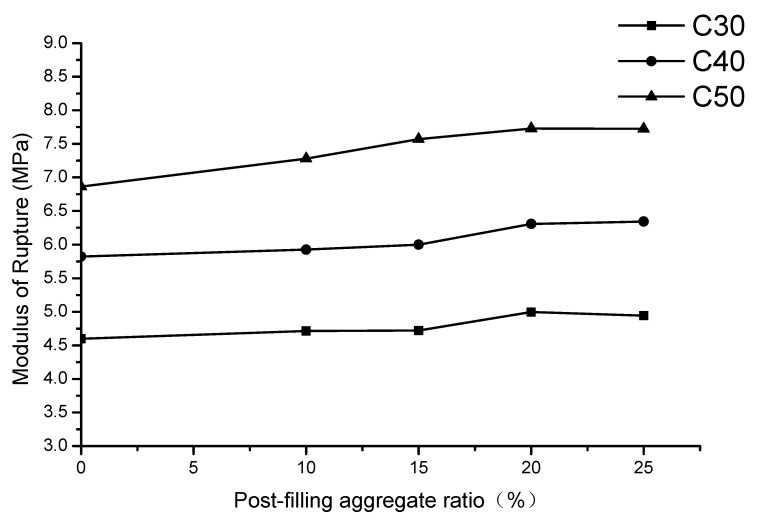
Relationship of modulus of rupture and post-filling coarse aggregate (PFA) ratio.

The modulus of ruptures (MORs) of tested specimens with variations of PFA ratios are shown in Table 6 and Figure 10. It can be found from Figure 10 that, the modulus of rupture of PFCC are improved compared with reference concrete for three strength grade concrete. For C30, the highest MOR is 5.0 MPa, which is increased by 0.4 MPa (8.7%) compared with reference concrete. For C40, the highest MOR reaches 6.34 MPa, which is improved by 0.52 MPa (8.9%) compared with the reference concrete. For C50, the highest MOR reached 7.73 MPa, which was 0.86 MPa (12.5%) higher than that of the reference concrete. It is also observed that the 20% PFA ratio is a turning point of MOR for C30 and C50. Additionally, the MORs of the C40 specimens increase with the increase of PFA ratios. It is recommended that the optimized PFA ratio is 15–20% for the sake of the modulus of rupture of concrete.

### 3.5. Modulus of Elasticity

**Figure 11:**
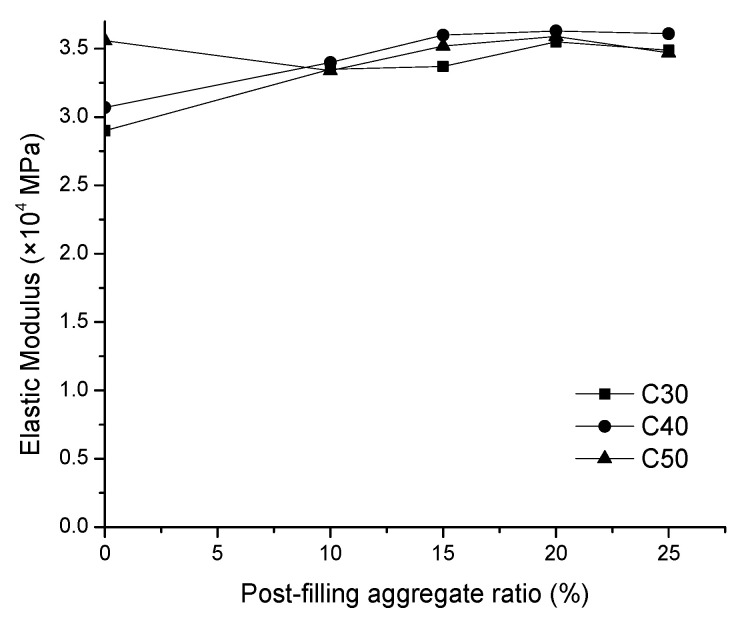
Relationship between modulus of elasticity and PFA ratio.

The modulus of elasticity of the concrete specimens with variations of the PFA ratio are shown in Figure 11. The relationship between the modulus of elasticity of concrete with its compressive strength as well as its apparent density can be found in the ACI (American Concrete Institute) building code as the following Equation (1).

(1) $$E_{c}=0.043\omega_{c}^{1.5}\sqrt{f_{c}^{\prime }}$$

where $E_{c}$ is secant line modulus of elasticity; $\omega_{c}$ is apparent density of concrete; $f_{c}^{\prime }$ is the compressive strength of concrete cylinder specimen (150 mm in diameter and 300 mm in length).

**Figure 12:**
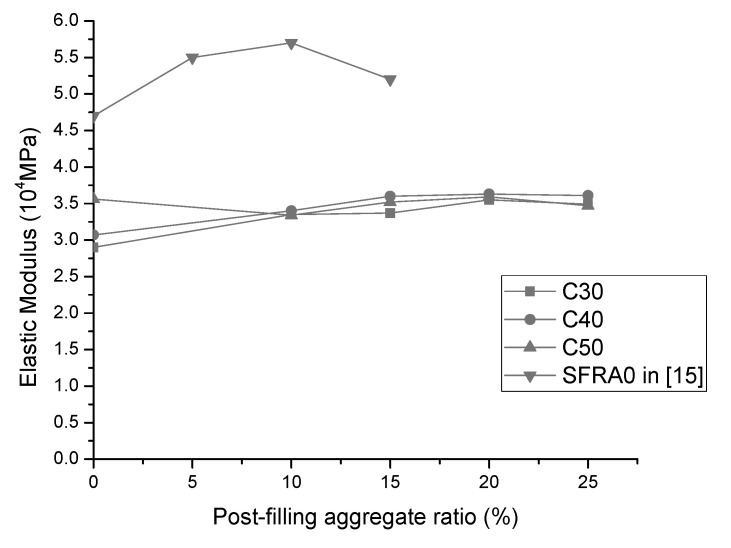
Comparison of elastic moduli in this study with literature results.

It can be seen from Figure 11 that the trend for the modulus of elasticity is similar as that of compressive strength. With the increase of the PFA ratio, the modulus of elasticity of PFCC increases first and then decreases, reaching the maximum modulus of elasticity at 20% PFA ratio for all concrete grades. This is consistent with the numerical model prediction in Equation (1). As the post-filling coarse aggregate ratio increases, the density of the concrete increases and it causes the modulus of elasticity of the PFCC increase. It shows that the highest modulus of elasticity of the C30, C40 and C50 PFCC reaches 3.55 × 10^4^, 3.63 × 10^4^ and 3.59 × 10^4^ MPa, respectively. The increase of the moduli of elasticity compared with the reference concrete are 0.65 × 10^4^ (22.4%), 0.56 × 10^4^ (18.2%) and 0.03 × 10^4^ MPa (0.84%). Generally, the modulus of elasticity of the PFCC is greater than that of the reference concrete. It is worthy to mention that, unexpectedly, the modulus of elasticity of the reference concrete is higher than that of the C50 PFCC at the 10% and 15% PFA ratio. This inconsistency is speculated to be caused by test errors and discreteness of concrete specimens. Figure 11 also indicates that at 25% PFA ratio, the modulus of elasticity begins to decrease. This is consistent with Equation (1) as the apparent density of concrete drops due to the segregation of concrete observed in the slump test. Meanwhile, Xu et al. [15] found the similar developing trend of elastic modulus for scatter-filling recycled coarse aggregate concrete. It demonstrated that the elastic modulus increased to the peak at 10% scattering-filling ratio and then it dropped at 15% scattering-filling ratio [15]. In addition, Figure 12 compares the elastic modulus results in this research with the literature findings. SFRA0 indicates the scattering filling coarse aggregate concrete with recycled coarse aggregate. It can be inferred that, similarly as C30 and C40 concrete, the elastic modulus of scattering filling coarse aggregate in [15] also rose to the maximum value at the 10% PFA ratio and then dropped. Although the turning point is slightly different, in general, the elastic moduli with post-filling aggregate are higher than those of the reference concrete. Overall, it is suggested that the optimized PFA ratio is 20%.

### 3.6. Axial Compressive Strength of Prism Specimen

The axial compressive strength of prism specimen with dimension of 150 mm × 150 mm × 300 mm was also tested. It was observed that the vertical cracks (parallel to the longitudinal direction) of the prism specimen appeared until the peak load was reached. Then, the load carrying capacity dropped immediately and the vertical cracks continued to develop toward the top and bottom of the specimen in width and in depth. Finally, the specimen was failed by the crushing of concrete and a penetrating diagonal crack was formed accompanied by the cracking sound.

**Figure 13:**
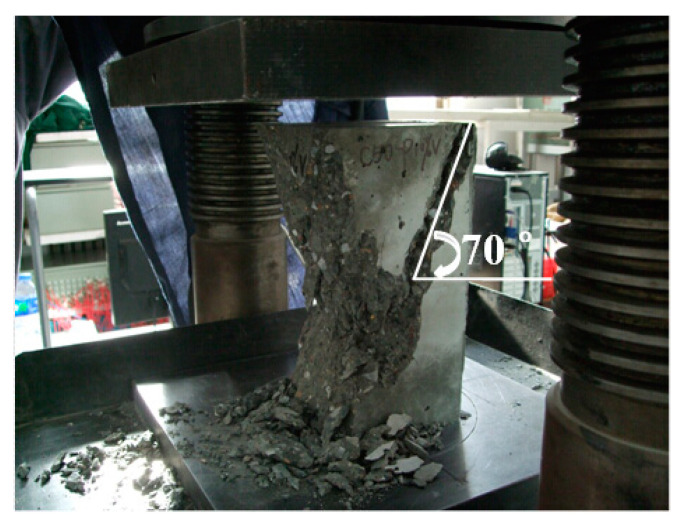
The failure mode of axial compressive test for PFCC.

In the axial compression test, the failure mode of the PFCC specimen is primarily the same as that of the reference concrete specimen, which are both brittle failure modes along the longitudinal direction. It exhibits that, as seen in Figure 13, the splitting crack is at a 70 degree angle (compared to the 50–60 degree of the reference concrete) to that of the horizontal plane. During the experiment, it was also found that the cracking sound of PFCC is larger than that of reference concrete, indicating that more coarse aggregate in the PFCC was split off than that in reference concrete.

**Figure 14:**
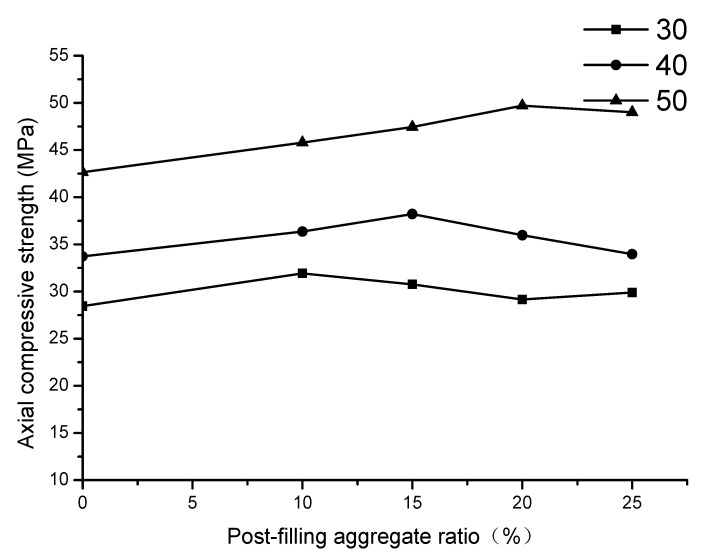
Relationship of PFA ratio and axial compressive strength.

The test results of the axial compressive strength of PFCC are shown in Table 7 and Figure 14.

As shown in Table 7, for C30, the highest axial compressive strength of PFCC is 31.9 MPa, which is increased by 3.4 MPa (11.9%) compared with the reference concrete. For C40, the highest axial compressive strength of PFCC reaches 38.2 MPa, which is increased by 4.5 MPa (13.4%) compared with the reference concrete. For C50 concrete, the highest axial compressive strength is 49.7 MPa and it is increased by 7.0 MPa (16.4%) compared with the reference concrete. It also exhibits that the highest strengths for C30, C40 and C50 grade concrete are located at the PFA ratios of 10–15%, 15%, and 20% respectively.

Therefore, it can be inferred that the axial compressive strength for the prism specimen increases and then decreases with the increase of the PFA ratio generally. The highest compressive strength occurs at the approximate PFA ratio of 10–20%. Compared with the reference concrete, the axial compressive strength of the PFCC is approximately increased by 10% overall. A 15–20% post-filling aggregate ratio is suggested for axial compressive strength.

### 3.7. Economic Cost Analysis of PFCC Materials

**Figure 15:**
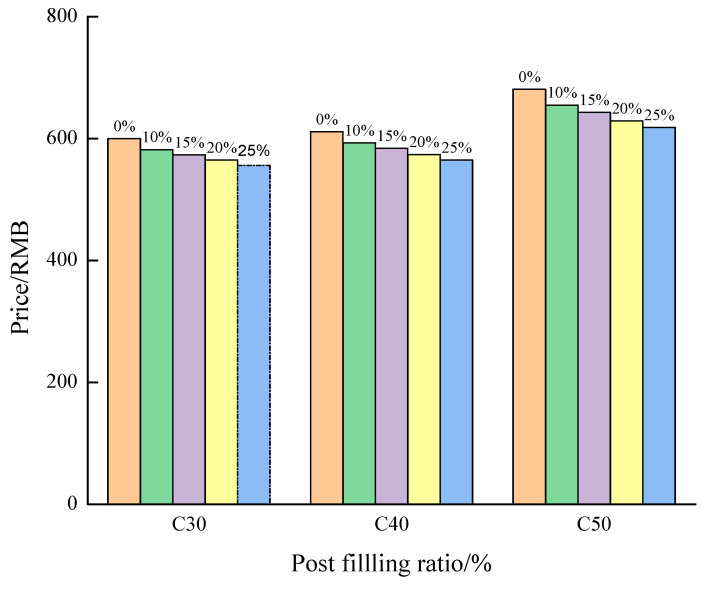
Effect of post-filling ratio on the cost of PFCC.

A cost analysis was conducted for the reference concrete and post-filling aggregate concrete at various post-filling ratio according to the current market price. The results are shown in Table 8, Table 9 and Table 10 and Figure 15.

It can be seen from Figure 15 that, as the post-filling ratio increases, the unit cost for post-filling concrete decreases. It also demonstrates that, as the concrete strength increases, the cost reduction decreases. It exhibits that the post-filling coarse aggregate concrete is fairly promising in saving the cost of concrete materials.

## 4. Conclusions

The objective of this research is to elucidate the relationship between the post-filling coarse aggregate technology, particular the post-filling ratios, and the mechanical properties of large slump PFCC associated with different concrete strength grades. It aims to provide a promising concrete production technology on the premise of its cost-saving as well as environmental benefits. The workability, compressive strength, elastic modulus, splitting tensile strength and modulus of rupture were experimentally tested and analyzed. Based on the experimental study and analysis of mechanical properties of the post-filling coarse aggregate concrete (PFCC), a few specific conclusions can be drawn.

(1) With the increase of the post-filling coarse aggregate (PFA) ratio, the slump of concrete decreases. Experimental results indicate that the slump of PFCC could meet the designed requirement of larger than 140 mm when the PFA ratio is no more than 15%.

(2) Results show that the failure mode of PFCC is similar as that of reference concrete. For C30 and C40 concrete, the failure of PFCC is basically the bond failure between the coarse aggregate and the cement paste. While the splitting cracking failure of the coarse aggregate was observed primarily for the C50 concrete. Moreover, the brittleness of the PFCC specimen is higher than that of the reference concrete specimen.

(3) The compressive strength test results of the cubic specimen show that the compressive strength of the PFCC at 7 d and 28 d increases first and then decreases as the PFA ratio increases for three concrete grades, which are all higher than those of reference concrete. For cubic compressive strength, the optimized PFA ratio in volume is 20%.

(4) The splitting tensile strength, modulus of rupture, axial compressive strength as well as the modulus of elasticity of PFCC are improved compared with those of reference concrete. The recommended PFA ratio is 15% for above properties. It was also found that, as the concrete strength grade increases, the enhancement of splitting tensile strength, modulus of rupture, axial compressive strength and modulus of elasticity in absolute value are increased.

(5) Generally, it does not indicate significant differences on the failure modes between the post-filling coarse aggregate concrete specimens and the reference concrete specimens.

## Figures and Tables

**Table 1:** Properties of polycarboxylate superplasticizer.

Water Reduction Efficiency	Matrix	Appearance	Specific Gravity	pH Value
30%	Modified polycarboxylate solution	Light brown	1.06–1.2	6–8

**Table 2:** Mixture proportions for three concrete grades.

Concrete Strength Grade	Cement + Fly Ash + Mineral Powder	Sand	Coarse Aggregate	Water	Water Reducer	Slump (mm)	7d Compressive Strength (MPa)	28d Compressive Strength (MPa)
C30	352 + 88 + 0	716	1074	220	4.4	200	21	32
	0.8:0.2:0	0.4	0.6	0.5	1%			
C40	352 + 44 + 44	716	1074	202	5.72	190	28	41
	0.8:0.1:0.1	0.4	0.6	0.46	1.3%			
C50	388 + 97 + 0	705	1058	194	7.28	200	41	54
	0.8:0.2:0	0.4	0.6	0.4	1.5%			

Note: The percentage of water reducer is based on the cementitious materials (cement and fly ash). The concrete mixture component unit is kg/m^3^.

**Table 3:** Specimen sizes.

Mechanical Property	Dimension
Compressive strength for cubic specimen ($f_{\mathrm{cu}}$)	150 mm × 150 mm × 150 mm
Compressive strength for prism specimen ($f_{c}$)	150 mm × 150 mm × 300 mm
Modulus of elasticity ($E_{c}$)	150 mm × 150 mm × 300 mm
Splitting tensile strength ($f_{\mathrm{ts}}$)	150 mm × 150 mm × 150 mm
Modulus of rupture ($f_{r}$)	150 mm × 150 mm × 700 mm

**Table 4:** Slump test results.

Concrete Grade	Slump (mm)				
	Post-Filling Ratio 0%	Post-Filling Ratio 10%	Post-Filling Ratio 15%	Post-Filling Ratio 20%	Post-Filling Ratio 25%
C30	200	150	145	120	110
C40	190	150	140	90	80
C50	220	170	150	115	90

**Table 5:** Compressive strength of cubic specimens.

Concrete Grade	Post-Filling Aggregate Ratio	Compressive Strength (MPa)	
		7d	28d
C30	0%	20.5 (1.3)	32.4 (1.4)
	10%	25.1 (1.1)	36.5 (1.9)
	15%	26.0 (1.2)	38.2 (2.4)
	20%	26.9 (0.4)	39.1 (3.6)
	25%	26.3 (1.5)	38.2 (4.5)
C40	0%	28.9 (1.3)	40.9 (0.7)
	10%	32.7 (3.0)	43.4 (0.3)
	15%	35.4 (2.0)	46.3 (0.3)
	20%	38.5 (2.2)	49.0 (2.8)
	25%	33.0 (1.6)	44.1 (0.2)
C50	0%	41.1 (0.4)	54.0 (2.8)
	10%	46.3 (1.0)	59.7 (3.1)
	15%	46.4 (1.7)	60.5 (0.8)
	20%	47.5 (0.2)	64.8 (0.7)
	25%	40.8 (1.1)	53.1 (3.1)

Note: Numbers in parenthesis indicate the standard deviation from the test results of three duplicate samples.

**Table 6:** Modulus of rupture.

Concrete Strength Grade	PFA Ratio	Modulus of Rupture (MPa)
C30	0%	4.60 (0.14)
	10%	4.71 (0.46)
	15%	4.72 (0.31)
	20%	5.00 (1.34)
	25%	4.94 (1.47)
C40	0%	5.82 (0.12)
	10%	5.93 (0.16)
	15%	6.00 (0.31)
	20%	6.31 (0.44)
	25%	6.34 (0.22)
C50	0%	6.87 (0.04)
	10%	7.28 (0.46)
	15%	7.57 (0.39)
	20%	7.73 (0.18)
	25%	7.72 (0.95)

Note: Numbers in parenthesis indicate the standard deviation from the test results of three duplicate samples.

**Table 7:** Axial compressive strength for prism specimen.

Concrete Strength	PFA Ratio	Average of Axial Compressive Strength (MPa)
C30	0%	28.5 (3.7)
	10%	31.9 (2.4)
	15%	30.8 (2.1)
	20%	29.2 (0.7)
	25%	29.9 (1.7)
C40	0%	33.7 (1.1)
	10%	36.4 (0.7)
	15%	38.2 (0.3)
	20%	36.0 (1.8)
	25%	34.0 (1.1)
C50	0%	42.7 (4.0)
	10%	45.8 (2.1)
	15%	47.4 (0.3)
	20%	49.7 (2.5)
	25%	49.0 (2.0)

Note: Numbers in parenthesis indicate the standard deviation from the test results of three duplicate samples.

**Table 8:** Cost comparison between reference and post-filling coarse aggregate concrete (C30).

Material		Cement	Fly Ash	CA	FA	Water	WR	Cost (RMB)/m^3^
Unit Price/(RMB·kg^−1^)		0.55	0.4	0.17	0.17	0.0025	15	
Reference concrete	Proportion	352	88	1074	716	220	4.4	599.65
	cost	193.6	35.2	182.58	121.72	0.55	66	
PFCC 10%	Proportion	316.8	79.2	1216.6	644.4	198	3.96	582.19
	cost	174.24	31.68	206.82	109.55	0.495	59.4	
PFCC 15%	Proportion	299.2	74.8	1287.9	608.6	187	3.74	573.45
	cost	164.56	29.92	218.94	103.46	0.468	56.1	
PFCC 20%	Proportion	281.6	70.4	1359.2	572.8	176	3.52	564.72
	cost	154.88	28.16	231.06	97.38	0.44	52.8	
PFCC 25%	Proportion	264	66	1430.5	537	165	3.3	555.99
	cost	145.2	26.4	243.19	91.29	0.41	49.5	

Note: the unit for materials proportion is kg/m^3^.

**Table 9:** Cost comparison between reference and post-filling coarse aggregate concrete (C40).

Material		Cement	Fly Ash	Mineral Powder	CA	FA	Water	WR	Cost (RMB)/m^3^
Unit Price/(RMB·kg^−1^)		0.55	0.12	0.5	0.17	0.17	0.0025	15	
Reference concrete	Proportion	352	44	44	1074	716	202	5.72	611.49
	Cost	193.6	5.28	22	182.58	121.72	0.51	85.8	
PFCC 10%	Proportion	316.8	39.6	39.6	1216.6	644.4	181.8	5.15	592.82
	Cost	174.2	4.75	19.8	206.82	109.55	0.45	77.25	
PFCC 15%	Proportion	299.2	37.4	37.4	1287.9	608.6	171.7	4.86	583.52
	Cost	164.6	4.49	18.7	218.94	103.46	0.43	72.9	
PFCC 20%	Quantity	281.6	35.2	35.2	1359.2	572.8	161.6	4.58	574.26
	Cost	154.9	4.22	17.6	231.06	97.38	0.4	68.7	
PFCC 25%	Proportion	264	33	33	1430.5	537	151.5	4.29	564.87
	Cost	145.2	3.96	16.5	243.19	91.29	0.38	64.35	

Note: the unit for materials proportion is kg/m^3^.

**Table 10:** Cost comparison between reference concrete and post-filling coarse aggregate concrete (C50).

Material		Cement	Fly Ash	CA	FA	Water	WR	Cost (RMB)/m^3^
Price/(RMB·kg^−1^)		0.6	0.4	0.17	0.17	0.0025	15	
Reference concrete	Quantity	388	97	1058	705	194	7.275	680.93
	Cost	232.8	38.8	179.86	119.85	0.49	109.13	
PFCC 10%	Quantity	349.2	87.3	1202.2	634.5	174.6	6.5	654.62
	Cost	209.52	34.92	204.37	107.87	0.44	97.5	
PFCC 15%	Quantity	329.8	82.5	1274.3	599.3	164.9	6.2	642.80
	Cost	197.88	33	216.63	101.88	0.41	93	
PFCC 20%	Quantity	310.4	77.6	1346.4	564	155.2	5.8	629.44
	Cost	186.24	31.04	228.89	95.88	0.39	87	
PFCC 25%	Quantity	291	72.8	1418.5	528.8	145.5	5.5	617.63
	Cost	174.6	29.12	241.15	89.90	0.36	82.5	

Note: the unit for materials proportion is kg/m^3^.
